# Genome-wide association study and genomic selection of flax powdery mildew in Xinjiang Province

**DOI:** 10.3389/fpls.2024.1403276

**Published:** 2024-05-28

**Authors:** Leilei Zhu, Gongze Li, Dongliang Guo, Xiao Li, Min Xue, Haixia Jiang, Qingcheng Yan, Fang Xie, Xuefei Ning, Liqiong Xie

**Affiliations:** ^1^ Xinjiang Key Laboratory of Biological Resources and Genetic Engineering, College of Life Science and Technology, Xinjiang University, Urumqi, China; ^2^ State Key Laboratory of Crop Stress Adaptation and Improvement, School of Life Sciences, Henan University, Zhengzhou, China; ^3^ Department of Basic Medicine, Xinjiang Second Medical College, Karamay, China; ^4^ Key Laboratory of Plant Stress Biology in Arid Land, College of Life Science, Xinjiang Normal University, Urumqi, China

**Keywords:** flax, powdery mildew (PM), quantitative trait loci (QTL), genome-wide association study (GWAS), genomic selection (GS)

## Abstract

Flax powdery mildew (PM), caused by *Oidium lini*, is a globally distributed fungal disease of flax, and seriously impairs its yield and quality. To data, only three resistance genes and a few putative quantitative trait loci (QTL) have been reported for flax PM resistance. To dissect the resistance mechanism against PM and identify resistant genetic regions, based on four years of phenotypic datasets (2017, 2019 to 2021), a genome-wide association study (GWAS) was performed on 200 flax core accessions using 674,074 SNPs and 7 models. A total of 434 unique quantitative trait nucleotides (QTNs) associated with 331 QTL were detected. Sixty-four loci shared in at least two datasets were found to be significant in haplotype analyses, and 20 of these sites were shared by multiple models. Simultaneously, a large-effect locus (*qDI 11.2*) was detected repeatedly, which was present in the mapping study of flax pasmo resistance loci. Oil flax had more QTL with positive-effect or favorable alleles (PQTL) and showed higher PM resistance than fiber flax, indicating that effects of these QTL were mainly additive. Furthermore, an excellent resistant variety C120 was identified and can be used to promote planting. Based on 331 QTLs identified through GWAS and the statistical model GBLUP, a genomic selection (GS) model related to flax PM resistance was constructed, and the prediction accuracy rate was 0.96. Our results provide valuable insights into the genetic basis of resistance and contribute to the advancement of breeding programs.

## Introduction

Flax (*Linum usitatissimum* L.) is an important oil and fiber crop around the world (Wu et al., 2017), divided into oil flax, fiber flax, and oil–fiber dual-purpose flax (OF). Oil flax is the ancestor of cultivated flax, and the OF is an evolutionary intermediate transition state between oil and fiber flax (Guo et al., 2020). Among the various threats to flax production, powdery mildew (PM) caused by *Oidium lini* Skoric is one of the most widespread and common diseases (Bengtsson et al., 2017; Duk et al., 2021). This fungal infection affects the growth of flax plants from vegetative stage to flowering. With the most severe impact occurring during ripening under conditions of high humidity and temperature (Stadlmeier et al., 2018), susceptible varieties can experience yield losses of up to 75% (Javid et al., 2015). Since 1970, the screening of accessions to PM resistance has been carried out in European countries. Scientists have conducted standard phenotypic identification to evaluate the resistance to PM in flax and screened PM resistance accessions (Rashid and Duguid, 2005; Dash et al., 2016; Dhirhi et al., 2017). However, only a limited number of resistant varieties have been identified and shown unstable resistance in different cultivation years (Aly et al., 2012; Hall et al., 2016). A similar circumstance exists in China (He et al., 2007; Qiao and Chen, 2012; Chen et al., 2018), where flax is an essential cash crop in several provinces with limited water resources (Zhou, 2020). There are many studies that have reported the unstable genotypic response of flax to PM across various ecological zones. For example, AC Watson shows moderate resistance in Canada (Rashid and Duguid, 2005), while it is susceptible in China (Qiao and Chen, 2012). Similarly, Diana exhibits a resistant response in the Indian climate (Wang et al., 2019) but is being used as a susceptible parent in the Heilongjiang flax breeding program for PM-resistant cultivar development (Zhang, 2015). It seems that the mechanism of PM infection in flax appears to be more complex than initially understood.

As compared with research on rice, wheat, and other crops, research on flax PM resistance has been relatively limited. Only three flax PM resistance genes have been reported, namely, *pm1* (Rashid and Duguid, 2005), *ol* (Singh et al., 1989), and *pm-linum* (Zhang, 2015), while their specific positions on the chromosome are still unknown. Based on linkage mapping, using 143 simple sequence repeat (SSR) markers in the F3 and F4 populations (NorMan×Linda), Asgarinia et al. (2013) obtained three flax PM resistance-related quantitative trait loci (QTLs) located in linkage groups 1, 7, and 9 (genetic distance between 6.7 and 25.2 cM), which can explain 97% of the variation. Similarly, 9801–1, a mutant line, was used as a resistant parent in a hybridization program with different susceptible parents and identified the candidate gene “*pm-linum*” located in the ChrNew02 chromosome (Yang et al., 2008; Zhang, 2015). Compared to linkage mapping, association mapping is more robust and has high accuracy, but its application in flax PM research is limited (Yano et al., 2016; Du et al., 2018). Based on phenotypic data of 311 flax accessions, Speck et al. (2022) performed a genome-wide association study (GWAS) using 1,693,910 single-nucleotide polymorphisms (SNPs) and MLM models and predicted eight QTLs across chromosomes 1, 2, 4, 13, and 14. You et al. (2022) performed GWAS on 5–8 years of phenotypic data from 447 flax accessions using 247,160 SNPs with nine models, and a total of 349 quantitative trait nucleotides (QTNs) were identified. The limited number of shared QTLs in existing studies suggested that this is highly quantitative and complex traits and demand to divulge underlying genetic mechanism.

GWAS is a method for analyzing genetic variation polymorphisms among varieties within a population at the genome-wide level (Li et al., 2021). It can screen and identify markers that are closely linked to target traits based on linkage disequilibrium (LD) (Lou et al., 2015) and reduce false-positive results by delineating a significance threshold. Large genomic datasets often exhibit a notable characteristic where SNPs that are physically close to each other often tend to display LD (Li et al., 2018), which is a major factor influencing marker density requirements and map resolution in GWAS analysis (Speck et al., 2022). This intricate high-dimensional and correlated structure within population genomic datasets poses difficulties for both single- and multi-locus models (Liang and Kelemen, 2008; Xu, 2013). Single-locus models rely on multiple detection corrections to reduce the occurrence of false positives. Bonferroni correction is the most traditional and extensively utilized approach (Dudbridge and Koeleman, 2004). However, this also makes the single-point model tend to identify locus with larger effects (Cui et al., 2018). A large number of studies have shown that most of the important economic traits of crops are influenced by polygenes, with contributing genes having a small effect on phenotype (Liu et al., 2017; Kang et al., 2020; Pogoda et al., 2020). The multi-locus mixed linear models, such as FASTmrEMMA (Wen et al., 2018), pLARmEB (Zhang et al., 2017), can effectively detect QTNs/QTLs with small effects and have been successfully applied in rape (Li et al., 2017), corn (Zhang et al., 2018), and other crops. Genomic selection (GS) is a promising breeding strategy that utilizes genome-wide markers to construct a statistical prediction model for obtaining genomic breeding values ​​of markers and haplotype effects (Meuwissen et al., 2013; Xu et al., 2020). By incorporating genetic variation from across the entire genome, GS can capture a broader range of relevant genetic information and increase selection accuracy and efficiency in the breeding process. The accuracy of the method has been demonstrated in plants such as maize, barley, and *Arabidopsis* (Lorenzana and Bernardo, 2009), and the findings obtained from GWAS can be effectively utilized in GS to enhance the accuracy and capabilities of genetic prediction models (Lan et al., 2020).

In this study, 200 flax core accession resources were evaluated for PM resistance in the field for 4 years in Yining, Xinjiang. GWAS was conducted utilizing 674,074 SNPs from this population to locate QTLs linked with PM resistance. Five multi-locus models and two single-locus models were used to identify resistance QTLs, and the results were compared with prior research to mine putative genes for PM resistance. At the same time, GWAS results were used to carry out GS, evaluate the efficiency of different markers in GS, and calculate breeding values, providing a theoretical basis for flax PM resistance breeding.

## Materials and methods

### Plant material

A diverse genetic panel of 200 cultivated flax accessions from the core collection was used (Guo et al., 2020). The core accessions were assembled from the world flax accession resources, collected from 41 countries and regions, corresponding to 11 geographical origins (**Supplementary Table S1**). This panel was grouped into three morphotypes: 71 oil flax, 51 fiber flax, and 78 OF.

### Powdery mildew resistance evaluation

The 200 accessions were evaluated for field PM resistance in the same nursery for 4 years, 2017 and 2019 to 2021, at Ili Kazakh Autonomous Prefecture Agricultural Science Institute, Xinjiang, China (43°55′N, 81°23′E; altitude, 681.6 m; the average July temperatures for 2017–2020 are 26.44°C, 26.27°C, 24.37°C, and 26.13°C, respectively, and their average humidity was 29.60%, 28.71%, 28.95%, and 28.89%, respectively). Each accession was seeded in 2-m rows spaced 40 cm apart during the second or third week of May every year. Spore suspension of PM from the last growing season was sprayed and inoculated to young shoots two to three times to ensure PM conidia dispersal and development. The disease index (DI) was calculated using the following formula:


$$\text{DI}=100\times \sum_{i=1}^{k}\left(\text{si}\times \text{ni}\right)/\left(5\times \text{N}\right),$$

where *i* is the *i*th disease score, *k* is the number of classes in the disease scale, *si* is the disease score (**Table 1**), *ni* is the number of plants at the corresponding disease score, and *N* is the total number of plants investigated (Willocquet et al., 2023). An accession was considered immune (I) when DI = 0, 0< DI< 20 highly resistant (HR), 20 ≤ DI< 40 resistant (R), 40 ≤ DI< 60 moderately resistant (MR), 60 ≤ DI< 80 susceptible (S), and DI ≥ 80 highly susceptible (HS) (Wang and Su, 2006). Field assessments were conducted at the early flowering stages (approximately July 20), and 30 normal plants of each variety were randomly selected to calculate the DI and used as phenotypic datasets for GWAS (**Supplementary Table S2**).

**Table 1:** Criteria for field assessment of powdery mildew on a scale of 0–5.

Disease score	Symptoms and degree of the disease
0	No sign of infection
1	Fewer than 1/3 leaves of the plant have been infected; the white powder is indistinct
2	1/3–2/3 leaves of the plant have been infected; the white powder is distinct
3	>2/3 leaves of the plant have been infected; the white powder is thickly formed together
4	The white powder grew thicker; the leaves started to turn yellow and necrotic
5	>2/3 leaves of the plant turned yellow and necrotic

### Genotyping and SNP identification

The sequencing and genotype calling, relative kinship, and LD analysis were performed as previously described (Guo et al., 2020).

### Genome-wide association study

GWAS analyses were conducted using the 4-year dataset and the 4-year average dataset with seven single-locus and multi-locus models (**Table 2**). Kinship genetic relationship matrices were estimated using the protocol suggested by each GWAS software package. The population structure of the 200 accessions was estimated using Frappe (http://med.stanford.edu/tanglab/software/frappe.html Supplementary). The principal component analysis (PCA) was performed using the MVP in the R package (https://github.com/XiaoleiLiuBio/MVP).

**Table 2:** Statistical methods used for GWAS.

Statistical model	Threshold for QTNs	GWAS software	References
GLM	−log(*p*) > 7.13	Tassel 5	(Price et al., 2006)
MLM	−log(*p*) > 5	Tassel 5	(Yu et al., 2006)
mrMLM	LOD > 3	mrMLM.GUI v4.0.2	(Zhang and Tamba, 2018)
FASTmrMLM	LOD > 3	mrMLM.GUI v4.0.2	(Zhang and Tamba, 2018)
FASTmrEMMA	LOD > 3	mrMLM.GUI v4.0.2	(Wen et al., 2018)
pLARmEB	LOD > 3	mrMLM.GUI v4.0.2	(Zhang et al., 2017)
ISIS EM-BLASSO	LOD > 3	mrMLM.GUI v4.0.2	(Tamba et al., 2017)

GWAS, genome-wide association study; QTNs, quantitative trait nucleotides.

The two single-point models were calculated using Tassel 5 software (**Table 2**), and the significant association threshold was determined using the Bonferroni-corrected critical *p*-value (α = 0.05). After correction, the selection threshold of GLM was −log_10_(*p*) = 7.13 (*p* = 0.05/674,074 SNPs), while the selection threshold of MLM was −log_10_(*p*) = 5. Five multi-locus models were performed using the R package mrMLM (**Table 2**). The selection threshold was logarithm of the odds (LOD) ≥ 3, which means that the rate of the existing target QTLs was 1,000 times higher than the probability that it does not exist.

After putative QTNs were identified, QTNs were grouped into QTLs. Based on LD analysis, 54 kb was the size of the linkage interval, and the obtained QTNs were grouped into QTLs by r^2^ (**Supplementary Figure S1**). The QTN with the highest average R^2^ across all datasets was chosen as a representative or tag for the QTLs (tag QTNs). Next, the significance of the DI differences was evaluated between two tag QTN alleles (henceforth referred to as the QTN effect) across all accessions. Wilcox non-parametric tests were performed using the R function *wilcox.test* (R Core Team, 2023) to remove the non-significant QTNs at a 5% probability level. Then, the resultant QTL set was filtered by selecting QTLs that were shared in at least two of the five datasets. Additionally, haplotype analysis was performed to keep QTLs with significant variations.

### Candidate gene prediction

Based on the maximum linkage interval (54 kb on either side of tag QTNs) and gene annotation using the *L. usitatissimum* genome’s GFF3 file, a total of 1,060 candidate genes were identified from 64 QTLs. Following functional annotation, disease resistance genes such as those with tetratricopeptide repeat (TPR), leucine-rich repeat (LRR), nucleotide-binding site (NBS), Toll/interleukin-1 receptor (TIR)–NBS–LRR (TNL), and receptor-like protein kinase (RLK) were selected. In order to find potential resistance genes, the discovered stable QTLs in this study were compared with those associated with disease resistance (Asgarinia et al., 2013; He et al., 2019a).

### Flax core collection evaluation

To investigate the QTL effect, correlations of the number of QTLs with positive-effect or favorable alleles (NPQTL) with DI in the five DI datasets were calculated using GraphPad Prism 8 (https://www.graphpad.com). Two hundred accessions were evaluated for their resistance to powdery mildew using the identified stable and significant QTLs. Two-dimensional clustering analysis of accessions and QTLs was conducted using the R package stats algorithm in the function “hclust” (R Core Team, 2023). The Euclidean distances between accessions or between QTLs were calculated based on QTL genotypes (“1” for positive-effect alleles and “2” for negative-effect alleles) using the “dist” function and the “Euclidean” method in R (R Core Team, 2023). Based on the DI score, 25 resistant (R) and 25 susceptible (S) extreme subgroups were chosen. The R package Complex heatmap was used to create a heatmap (Gu et al., 2016).

### Genomic selection

To improve the accuracy of genomic prediction models, using the R package lmerTest (Kuznetsova et al., 2017), the best linear unbiased predictor (BLUP) values (Robinson, 1991) were calculated as the phenotypic value based on 4 years’ data. The predictive ability of five marker sets was evaluated: 1) randomly selected from 674,074 single-nucleotide diversities, 2) QTNs identified by GWAS using all 674,074 SNPs, 3) QTLs that were shared across multiple datasets and were significant in haplotype analysis, 4) QTLs shared by multiple models, and 5) QTLs with large effect.

GS utilized two models—GBLUP (Ikeogu et al., 2019) (based on the SNP relationship matrix) and rrBLUP (Ahmadi et al., 2021) (based on the individual relationship matrix)—implemented using the R software package sommer (Covarrubias-Pazaran, 2016). The correlation between the phenotypic data of the predicted population and genomic estimated breeding values (GEBVs) based on fivefold cross-validation was calculated to analyze the accuracy of the prediction.

## Results

### Powdery mildew resistance evaluation

**Figure 1:**
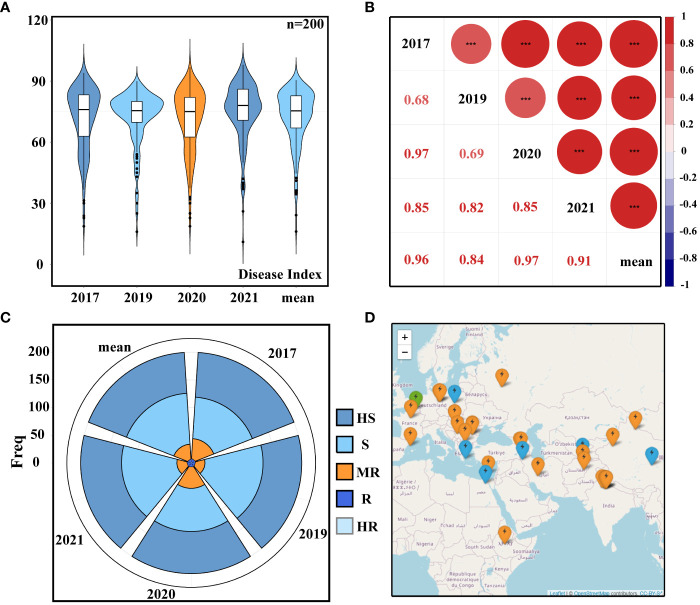
Resistance phenotypes. **(A)** Disease index (DI). **(B)** Correlation analysis of DI. **(C)** Classification of powdery mildew (PM) resistance: HS, high susceptible; S, susceptible; MR, moderately resistant; R, resistant; HR, high resistant. **(D)** Geographical distribution of resistant varieties: green represents highly resistant varieties, blue represents moderately resistant varieties, and orange represents resistant varieties. Different letters denote significant differences at the *p*< 0.05 level found by MRT. *** p<0.001.

A total of 200 accessions from 44 different countries were tested for PM resistance in the field trials during 2017, 2019, 2020, and 2021. The broad-sense heritability over the 4 years was calculated to be 0.93, indicating that the phenotype was mainly affected by genetic factors. The average DI recorded during these years was 72.6 (**Figure 1A**; **Supplementary Table S3**), and there were no significant differences in PM resistance. The five DI datasets’ correlation analysis revealed a highly substantial positive correlation between the five groups of data (**Figure 1B**), demonstrating that the PM resistance situation was relatively stable for 4 years and that the repeatability of the data was high. Furthermore, statistics was conducted on the PM resistance grading, and the 200 accessions were divided into five resistance groups based on the DI (**Figure 1C**; **Supplementary Table S4**). The geographical distribution of resistant cultivars across datasets showed that resistant accessions were mainly distributed in the Mediterranean coastal area and parts of Europe (**Figure 1D**).

### A total of 331 QTL identifications through GWAS

Based on 4 years of phenotypic datasets and the 4-year average dataset from 200 flax accessions, GWAS analysis was performed using 674,074 SNPs, two single-locus models, and five multi-locus models. A total of 443 QTNs were detected. The maximum linkage interval calculated using the LD decay plot was in the range of 54 kb (**Supplementary Figure S1**), and 434 QTNs were combined into 331 QTLs (**Supplementary Table S5**). QTNs with the highest average R^2^ were chosen as the tag to represent these QTLs (tag QTNs). Hereafter, the QTLs will be represented by the tag QTNs. In the two single-locus models, GLM identified 34 QTLs with an average contribution rate of 17.44%, which was higher than the average contribution rate identified by the MLM model (**Table 3**). Among the five multi-locus models, FASTmrMLM identified the highest number of QTLs (101) or QTNs (105), with an average R^2^ of 2.73%. The QTLs identified by mrMLM had the highest contribution rate, with an average R^2^ of 4.32% (**Table 3**). This result demonstrated that compared to single-locus models, the multi-locus models are more likely to detect QTLs with smaller effects. Only a small fraction of the total QTLs were found by at least two models (**Supplementary Table S6**). The pLARmEB and FASTmrMLM models identified the highest number of QTLs, amounting to 40, while the ISIS EM-BLASSO and pLARmEB models detected 26 loci.

**Table 3:** Comparison of QTN/QTL identifications for different statistical models.

Statistical model	No. of QTLs identified	No. of QTNs identified	Average R^2^ (%)	R^2^ range (%)
GLM	34	39	17.44	12.82–22.03
MLM	101	144	16.13	10.67–23.64
mrMLM	88	91	4.32	0.73–13.53
FASTmrMLM	101	105	2.73	0.08– 12.29
FASTmrEMMA	49	51	2.37	0.67–5.45
pLARmEB	94	101	1.95	0.11–11.33
ISIS EM-BLASSO	58	60	4.09	0.37–17.16

QTN, quantitative trait nucleotide; QTL, quantitative trait locus.

### Identification of QTLs across years and models

**Figure 2:**
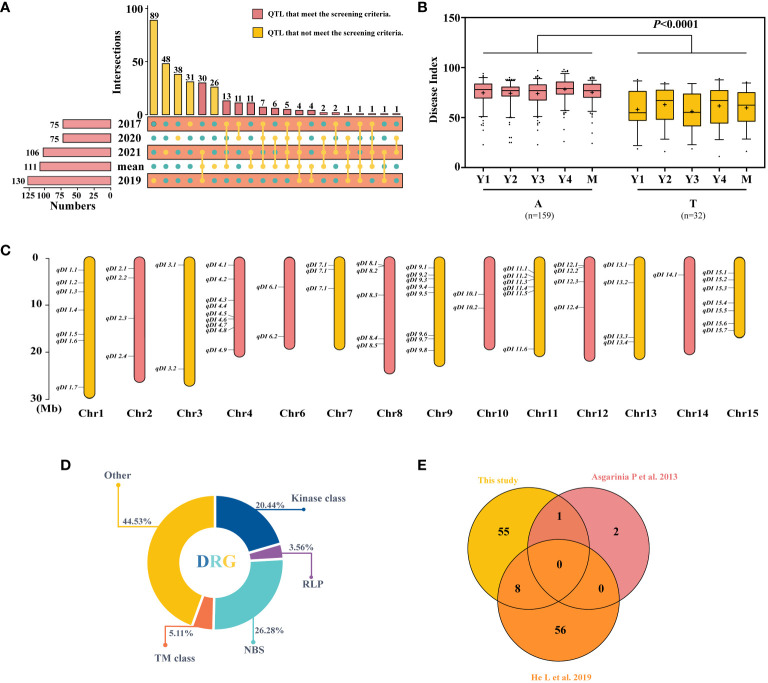
Screening and comparison of the obtained quantitative trait loci (QTLs) and candidate gene. **(A)** Repeated detection of QTL screening in different years. **(B)** Haplotype analysis of SNP15090, Y1–Y4, and M representing the 2017, 2019, 2020, 2021, and average datasets. **(C)** The distribution of 64 stable resistance loci on chromosomes. **(D)** Class distribution of disease resistance-related genes (DRGs) located within 54-kb flanking regions of QTLs. **(E)** Duplication between the resistance loci utilized in this investigation and those used in previous studies. The difference between haplotypes was analyzed by *t*-tests.

To explore the candidate QTLs for PM resistance, the 331 QTLs explained earlier were explored, and the 100 QTLs that had been identified in at least two datasets were selected (**Figure 2A**; **Supplementary Tables S7**, **S8**) and subjected to haplotype analysis. Using SNP15090 (tag QTNs) as an example, the locus with significant haplotype analysis among the resistant and susceptible variations was kept (**Figure 2B**). A total of 64 loci were identified and distributed across 14 chromosomes (**Figure 2C**; **Supplementary Table S9**). Similarly, 20 of these sites were shared by two single-site models or by more than three multi-site models simultaneously (**Supplementary Figure S2**). Due to the greater number of variants found in the 64 QTLs, we have chosen these 64 QTLs for further investigation in our following research.

Among the 64 stable QTLs, 49 harbored 137 disease resistance-related genes (DRGs) within the maximally linked regions of the QTNs (**Figure 2D**), encompassing five types of resistance gene analogs (RGAs): 1) kinases: protein kinase (PK), RLK, mitogen-activated protein (MAP), pyruvate kinase (PAK), and serine/threonine kinase (STK); 2) receptor-like protein (RLP); 3) NBS encoding genes: TNL, LRR, RNA binding protein (RBP), and sequence-specific transcription factors (TFs); 4) transmembrane proteins (TM classes): transmembrane receptor (TMR), amino acid transporter (AAT), transmembrane proteins (TMPs), Mildew Locus O (MLO), and transmembrane kinase (TMK); and 5) others: TPR, zinc finger protein (ZFP), alpha/beta hydrolases (ABHs), major latex protein (MLP), calcium-dependent lipid-binding (CalB), heat shock protein (HSP), abscisic acid (ABA), disease resistance-responsive (dirigent-like protein) family protein, nuclear transport factor (NTF), nucleotidyl transferase (NTase), nucleotide sugar transporter (NST), protein methyltransferase (PMT), peptide-*N*_4_-(*N*-acetyl-beta-glucosaminyl)asparagine amidase A (PNGase A), and GAI‐RGA‐and‐SCR family (GRAS). Most DRGs belong to the NBS gene family, accounting for 26.28%, followed by kinases, accounting for 20.44%. Additionally, we compared these findings to previous studies on flax resistance and found that some of the 64 QTLs reported in this study had already been reported (**Figure 2E**) (Asgarinia et al., 2013; He et al., 2019a). Asgarinia et al. (2013) mapped flax PM resistance genes to three linkage groups—LG1, LG7, and LG9—in 2013 and identified three loci. Comparatively, our study also identified the locus on LG7 (*qDI 3.2*), where a total of six resistance candidate genes were localized. There are also similarities in plant resistance to fungal diseases, most of which are common. By comparing with flax pasmo resistance loci, eight QTLs (*qDI 4.7*, *4.8*, *8.4*, *8.5*, *9.6*, *9.8*, *11.2*, and *12.1*) were repeatedly detected, along with 17 resistance candidate genes. In summary, after further screening, 64 stable QTLs were obtained, which comprise 137 DRGs. Of these 64 QTLs, 20 showed significant effects. Moreover, the results of the comparison with previous studies also demonstrate the reliability of the identified QTLs.

### Large-effect locus detected repeatedly—*qDI 11.2*

**Figure 3:**
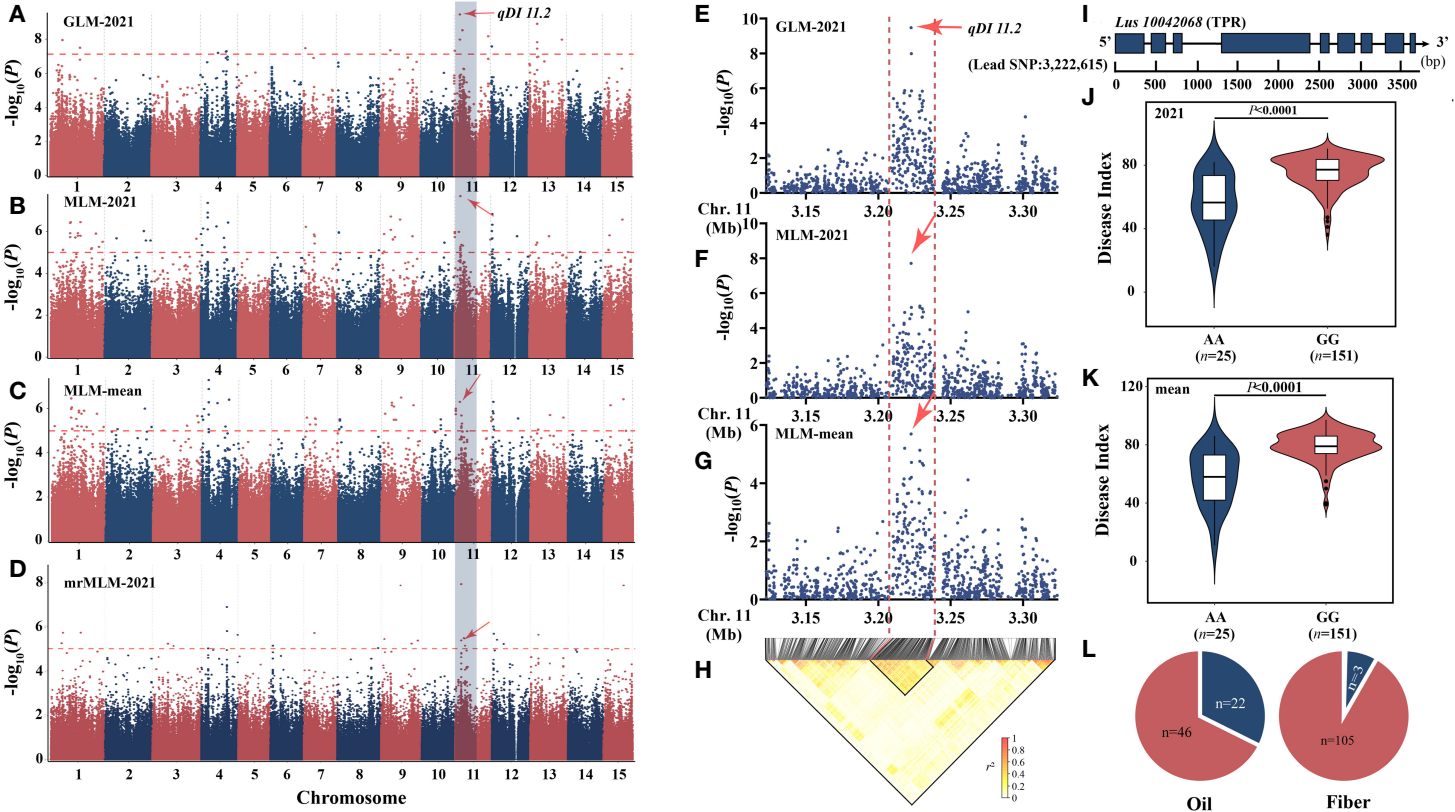
Analysis of the peak for chromosome 11 and candidate genes. **(A–D)** Manhattan plots based on GLM-2021 **(A)**, MLM-2021 **(B)**, MLM-mean **(C)**, and mrMLM-2021 **(D)**. **(E, F)** Local Manhattan plot surrounding the peak on chromosome 11. **(E)** GLM-2021. **(F)** MLM-mean. **(G)** MLM-mean. **(H)** LD heatmap. **(I)** Gene structure of *Lus10042068*. **(J, K)** Haplotype analysis based on the lead single-nucleotide polymorphism (SNP) (SNP450497) of 2021 **(J)** and mean **(K)**. **(L)** The distribution of allele frequencies of strong SNP was distributed in oil and fiber subpopulations. The AA and GG alleles are shown in blue and red, respectively. The difference between haplotypes was analyzed by *t*-tests.

Based on the aforementioned research, we discovered that the chromosome 11 locus *qDI 11.2* (R^2^ = 22.24) was commonly detected by GLM [−log_10_(*p*) = 9.47], MLM [2021: −log_10_(*p*) = 7.71, mean: −log_10_(*p*) = 5.69], and mrMLM [−log_10_(*p*) = 11.00, LOD = 10.06] (**Figures 3A–D**) and was also observed by You’s lab (He et al., 2019a). The candidate interval was reduced to 30 kb by combining the local Manhattan map and the LD heatmap (**Figures 3E–H**). This region contains only one gene, *Lus10042068* (**Figure 3I**), which encodes TPR protein and has been involved in resistance to both bacterial blight and rice blast (Yang et al., 2019). Haplotype analysis showed that the AA genotype had a positive effect on flax PM resistance (**Figures 3J, K**). The AA genotype accounts for 32.4% of oil and 2.7% of fiber (**Figure 3L**).

### Positive effects of favorable alleles have additive effects

**Figure 4:**
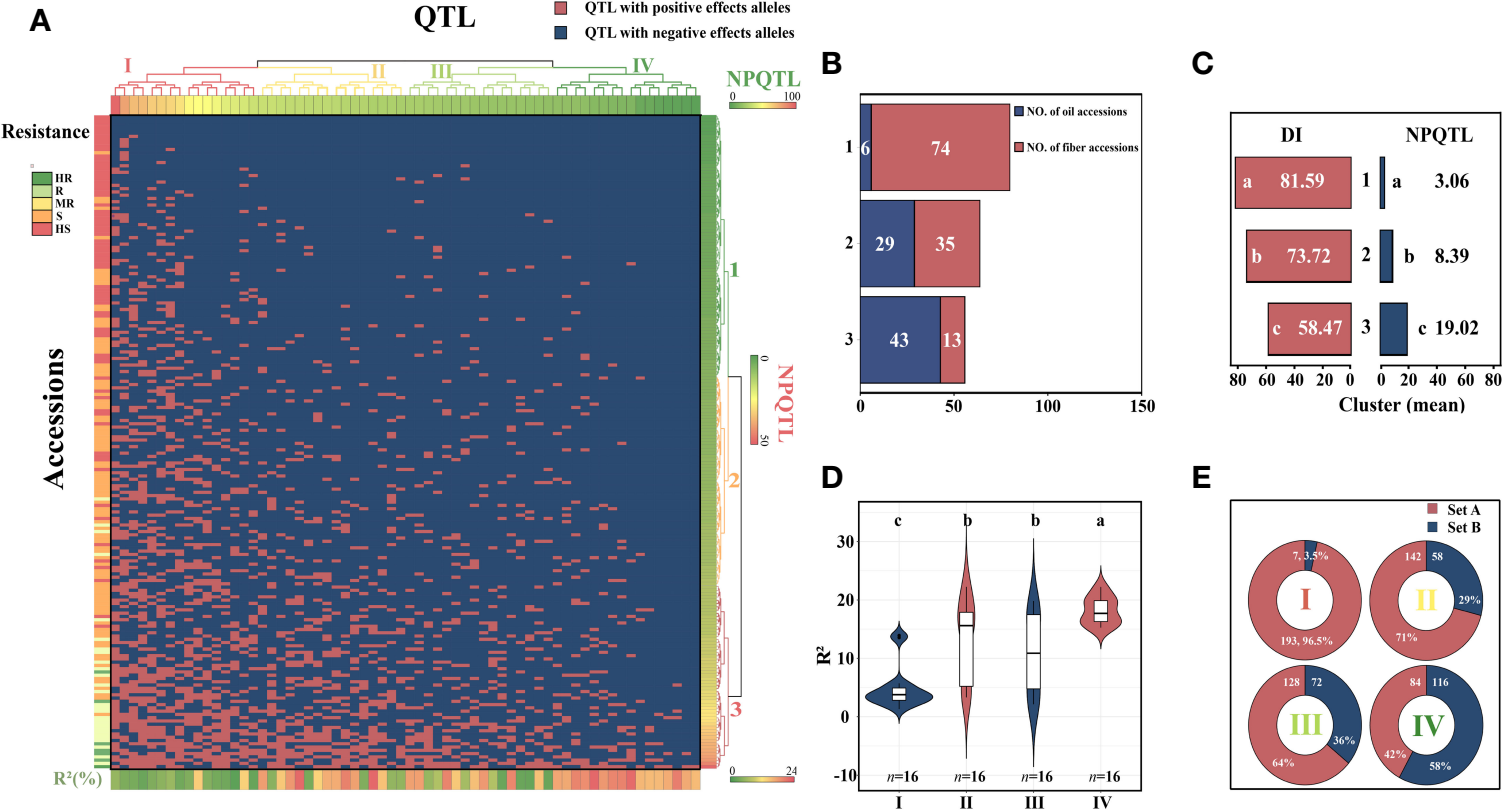
Cluster analysis of the association panel based on a set of 64 stable large-effect quantitative trait loci (QTLs). **(A)** The accessions were grouped into three clusters, and the QTLs were assigned to four subgroups. Tag QTNs of QTLs were chosen for analysis. Red indicates the presence of positive-effect or favorable alleles (PQTLs) in the accessions; blue indicates the absence of PQTLs. **(B)** The number of oil and fiber materials included in the materials clustered into three clusters. **(C)** DI, disease index; NPQTL, the number of QTLs with positive-effect alleles. **(D)** Violin plot of QTLs clustered into four groups. **(E)** Set A, the number of materials that contain this group of QTLs; Set B, number of materials excluding this group of QTLs. Different letters denote significant differences at the *p*< 0.05 level found by MRT.

The study of the correlation between the number of QTLs with NPQTL and DI revealed that in five datasets, both showed a highly significant negative correlation (r = −0.48–0.71, *p*< 0.0001), with the mean dataset exhibiting the strongest correlation (r = −0.71; *p*< 0.0001) (**Supplementary Figure S3**). In order to investigate the relationship between NPQTL and DI in the 200 core accessions, based on 64 core QTLs associated with PM resistance, a two-dimensional cluster analysis was carried out utilizing the tag QTNs as the representative of QTLs. The 200 accessions were divided into three clusters (**Figure 4A**). Cluster 1 contained 80 cultivars, showing strong susceptibility (DI = 81.6 ± 5.2), with an average of only 3.06 QTLs with positive-effect or favorable alleles (PQTLs) per cultivar (**Figures 4B, C**). Cluster 2 included four moderately resistant varieties, 44 susceptible varieties, and 16 highly susceptible varieties (DI = 73.7 ± 8.1) (**Figures 4B, C**). The majority of the accessions in Cluster 1 and nearly half of the accessions in Cluster 2 belonged to flax for fiber. The cultivars in Cluster 3 were the most resistant (DI = 58.5 ± 14.7), with an average of 19.02 PQTLs per cultivar, 43 PQTLs for oil, and 13 PQTLs for fiber (**Figures 4B, C**).

The 64 QTLs were clustered into four subgroups based on the QTL distribution in 200 accessions. The first group included 16 QTLs that were widely distributed among accessions but had relatively low QTL effects (**Figures 4D, E**). Group II contained 16 QTLs, with an average R^2^ of 13.48%, and was present in 71.0% of the accessions. Group III contained 16 QTLs, and 64.0% of the accessions had these QTLs, with an average R^2^ of 10.79%. The 16 QTLs in subgroup IV were mainly distributed in the resistant accessions, accounting for only 42% of the accessions, most of them were oil accessions, and the average R^2^ was 18.16% (**Figures 4D, E**). The highly resistant material C120 contains 41 resistance loci, and these excellent resistant accessions containing more resistance loci were very important for flax PM resistance breeding.

### Morphotype response to flax PM

**Figure 5:**
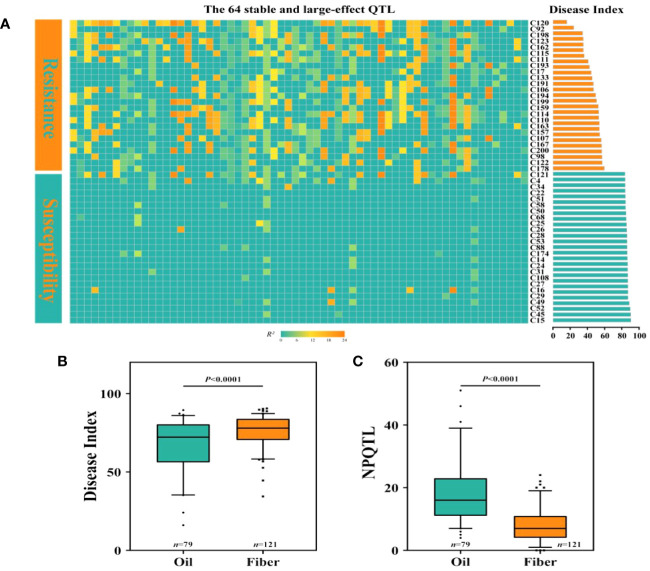
Distribution of number of quantitative trait loci (QTLs) with positive-effect or favorable alleles (NPQTL) in flax accessions. **(A)** Heatmap of disease index and NPQTL in resistant and susceptible individuals. **(B)** Comparison of disease index (DI) of oil and fiber in flax. **(C)** Comparison of NPQTL of oil and fiber in flax. The difference between subpopulations was analyzed by *t*-tests.

We discovered that among the 200 accessions of flax, the DI distribution of oil flax was significantly lower than that of fiber flax (**Figure 5B**). By comparing the DI of flax and the NPQTL contained in different flax accessions, we found a highly significant correlation between NPQTL and the flax subgroup (*r* = 0.34, *p*< 0.0001) (**Figures 5B, C**). For further analysis, we assigned each QTL a value of “1” for positive-effect alleles and “2” for negative-effect alleles. Chi-square test results showed that most of the PQTLs were significantly associated with oil accessions (**Figure 5C**) and that 80% to 100% of the PQTLs were present in oil varieties. Among the 200 flax accessions, NPQTL in different accessions ranged from 0 to 41. Then, we analyzed the aggregation of the PQTLs in two extreme subsets of flax accessions, consisting of 25 resistant (R) and 25 susceptible (S) accessions. Among them, 25 resistant accessions belonged to oil flax, and 25 susceptible accessions belonged to fiber flax. The mean DI in the R group was 45.8, each variety contained an average of 24.4 PQTLs, and they show stable resistance in many years of phenotypic data (**Supplementary Figure S4**). Among them, C120 showed high resistance in the 4-year resistance identification, and it had relatively good agronomic traits (**Supplementary Figure S4**, **Supplementary Table S10**); the mean DI of group S was 84.0, and each variety contained an average of 3.52 PQTLs (**Figure 5A**).

### Genomic selection

In order to improve the breeding efficiency of flax PM resistance, we used 4 years of field phenotype data to calculate the BLUP values, and a fivefold cross-validation scheme was used to identify the best models for GS of PM resistance. Five marker sets were used to construct the GS: 1) SNP-6741 was randomly selected from 674,074 single-nucleotide diversities. 2) SNP-331QTL has a total of 331 QTLs associated with PM resistance obtained based on the GWAS results. 3) SNP-64QTL was in the SNP-331QTL based on the selection of the set that was shared at least two times or more in the five DI sets and the significant loci of the haplotype analysis. 4) SNP-20QTL was selected based on the SNP-64QTL to be repeatedly detected by GLM and MLM, or by five, the multi-locus model repeatedly detects the locus set at least three times. 5) SNP-12QTL was a locus set with an effect value of more than 15% selected on the basis of SNP-64QTL.

**Figure 6:**
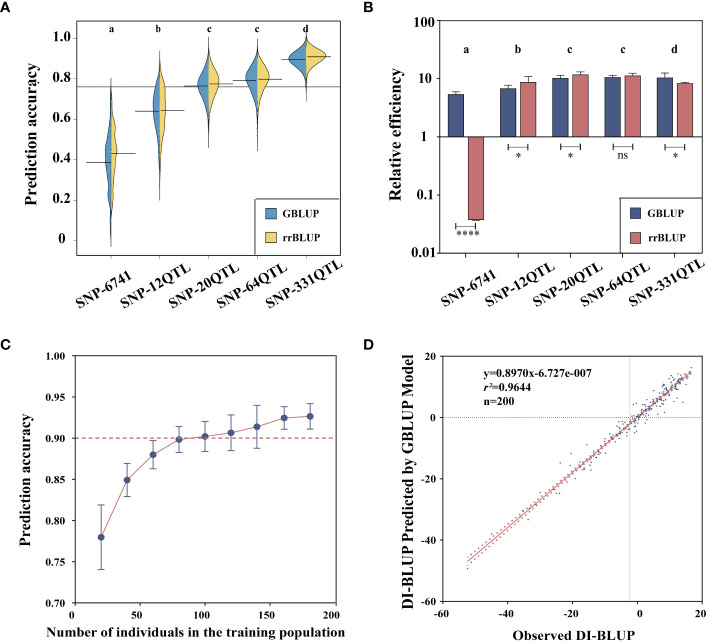
Genomic selection for flax powdery mildew. **(A)** Prediction accuracy of GBLUP and rrBLUP. **(B)** Relative efficiency of GBLUP and rrBLUP. **(C)** Correlation between training population size and prediction accuracy. **(D)** Correlation between observed and predicted values. Different letters denote significant differences at the *p*< 0.05 level found by MRT. * p<0.05, **** p<0.0001, ns: no significant.

Based on the SNP relationship matrix and the individual relationship matrix, respectively, we selected the GBLUP model and the rrBLUP model in GS prediction. These two models have much shorter running times while still maintaining prediction accuracy. First, we examined the two models’ accuracy when performed on various QTL datasets (**Figure 6A**). Accuracy based on the SNP-331QTL operation was the highest (0.92 ± 0.01 by GBLUP and 0.93 ± 0.01 by rrBLUP). The result based on the SNP-6741 operation had the lowest accuracy (0.39 ± 0.14 by GBLUP and 041 ± 0.13 by rrBLUP). The accuracy of the rrBLUP model was higher than that of the GBLUP model when calculations were based on the same dataset. However, the rrBLUP model’s efficiency was lower than the GBLUP model’s efficiency due to a significant increase in calculation time when the computation was based on a large number of datasets (**Figure 6B**). Taking all relevant factors into account, we will carry out further analysis based on the SNP-331QTL dataset and the GBLUP model. We also discovered that the prediction accuracy was promoted with the increasing number of training populations (**Figure 6C**). Finally, we developed a GS model with a 0.96 prediction accuracy, and the linear relationship was expressed as y = 0.8970x − 6.727e−007 (**Figure 6D**).

## Discussion

### Evaluation of flax powdery mildew resistance

In this study, we observed that the resistance to PM of oil cultivars was higher than that of fiber cultivars, the resistance population contained more PQTLs (**Figures 5B, C**), and the PM resistance of flax cultivars tended to increase with the NPQTL (**Figure 5A**) in its genome. The result aligns with the results obtained in Canada, where 5 years of continuous field phenotypic data were analyzed (You et al., 2022). At present, the view of the mainstream is that the ancestor of the existing flax varieties is *Linum bienne*, and the genetic diversity of oil flax is more abundant than that of fiber flax (Allaby et al., 2005; Fu and Allaby, 2010; Fu, 2011). Fiber materials are less resistant than oil materials, which may be attributed to the fact that resistance to PM was not the main selection criterion during the early human selection process. In addition, the presence of different physiological races of PM in various regions may result in variations in the resistance response of the same plant material across those regions (He et al., 2007; Qiao and Chen, 2012). We utilized the saturation inoculation method to evaluate the PM resistance in flax accessions because, in a natural environment, all susceptible flax plants may not be infected by pathogenic fungi at the same time, leading to significant variations in resistance identification of the same variety in the field. The Belgian cultivar C120 identified in this study showed stable high resistance to flax PM over many years of field observations, with 4-year DI grades ranging from 11 to 18.67. The cultivar L. 270–68 identified in this study showed stably high susceptibility to flax PM over many years of field observations, with 4-year DI grades ranging from 88 to 92 (**Figure 4A**). Smaller variations of DI indicated that the phenotype obtained in our study through saturation inoculation was stable and reliable. Therefore, we believe that the Belgian cultivar C120 with large numbers of 41 PQTLs was an excellent parent, which can be directly crossed with excellent flax varieties to improve the disease resistance of flax.

### Identification of QTLs

GWAS is widely used because it can screen multiple SNPs associated with complex trait variations on a genome-wide scale, and its core is the selection of algorithms, that is, analysis models. The commonly used single-locus models like GLM and MLM tend to identify QTNs or QTLs with larger effects (He et al., 2019b; Guo et al., 2020). However, most quantitative traits are controlled by a few high-influencing genes and many small-influencing polygenes (Arojju et al., 2020). To overcome this problem, we introduced multiple multi-locus models in this study to detect these loci with smaller effects (Gunjača et al., 2021). The results showed that in the single-locus models, fewer sites were detected by GLM, while more sites were detected by MLM, which may be due to the stricter correction threshold used by GLM. In the multi-locus models, FASTmrMLM detected the largest loci, while the mrMLM had the highest R^2^ (**Table 3**). Additionally, we observed that there were few shared loci between the single-point model and the multiple-point model (**Supplementary Table S6**). Therefore, the combination and complementarity of different models were very crucial.

Using 4-year flax PM datasets and 674,074 SNPs for GWAS, a total of 434 QTNs were detected based on two single-locus models and five multi-locus models. Optimal window size can be determined based on link imbalance decay (Remington et al., 2001; Sun et al., 2016). A maximum link interval of 54 kb was selected (**Supplementary Figure S1**), and 434 QTNs were merged into 331 QTLs. Additionally, through haplotype analysis, 64 loci with significant haplotype analysis were obtained, and 49 of them were co-localized with 137 DRGs (**Figure 2D**). Moreover, it was observed that 20 QTLs were consistently detected by multiple models, indicating their robustness and reliability (**Supplementary Figure S2**). By comparing with previous literature (Asgarinia et al., 2013; He et al., 2019a; Speck et al., 2022; You et al., 2022), a total of 17 identical QTLs were obtained. Encouragingly, certain loci, such as *qDI 4.7* and *qDI 8.4*, had been identified consistently across multiple studies. This indicated that these shared loci were highly stable. These QTLs provide important information for further dissecting the mechanisms and breeding of powdery mildew-resistant flax varieties. By leveraging these genetic markers, breeders can expedite the process of selecting and developing resistant cultivars, thereby advancing sustainable flax production and bolstering crop health in the presence of powdery mildew challenges.

### Flax mildew resistance candidate gene *Lus10042068*

According to our research, the *qDI 11.2* (R^2^ = 22.24) was a large-effect locus (**Figure 3**); shared by GLM, MLM, and mrMLM models; and also detected in the flax pasmo resistance locus mapping study (He et al., 2019a). The candidate range was further narrowed by combining the LD map with the local Manhattan map, and the *Lus10042068* gene was located. The functional region of this gene encodes a tetrapeptide repeat superfamily protein, whose homologous gene in rice has been proven to be a resistance gene to blast and bacterial blight by means of overexpression and knockout (Yang et al., 2019). The gene is regulated by reactive oxygen species metabolism in response to the infection of *Magnaporthe grisea*, not only as an important node in the effector-triggered immunity (ETI) process but also in response to the pattern-triggered immunity (PTI)-related immune signaling pathway triggered by PAMP. This is also consistent with the major breakthrough in the principle of interaction between PTI and ETI in recent years, so *Lus10042068* in flax may also play an important regulatory role in the process of plant PTI and ETI.

### Genomic selection

In this study, four QTL marker sets associated with PM (SNP-331 QTL, SNP-64 QTL, SNP-20 QTL, and SNP-12 QTL) and a set of genome-wide SNP markers (SNP-6741) were used to evaluate flax population breeding value. Our findings demonstrated that the SNP-331 QTL-based GS model was consistently superior to the models made with the other four marker sets. The robustness and reliability of QTLs identified by unit point and multi-locus GWAS statistical models were verified. All or virtually all PM-related QTLs were present in the SNP-331 QTL, and additional markers instead reduced prediction accuracy. Likewise, further screening of these QTLs with stricter criteria would reduce accuracy. Our results further demonstrated why QTLs with high effects sometimes fail to fully account for genetic traits in quantitative trait-related gene mapping (Jighly et al., 2019). Previous studies have also shown that the use of genome-wide QTN/QTL markers instead of random SNP markers for GS model construction can improve the prediction accuracy of GS (He et al., 2019a; Lan et al., 2020). Therefore, it is desirable to create a GS model including all potential QTLs linked to the chosen trait because it considerably increases prediction accuracy. Similar findings have been found for the related genome-wide predictions of flax blight (He et al., 2019b).

## Conclusion

We conducted a 4-year field evaluation for PM resistance of 200 flax accessions collected from 44 countries. The data demonstrated high reproducibility, which increased the credibility of the study’s findings. Using a genome-wide high density of SNPs, combined with multiple single-locus and multi-locus models, we identified 64 QTL and 137 resistance candidate genes and demonstrated the importance of combining multiple models. At the same time, we identified a repeatedly detected QTL with a large effect, and we identified a candidate gene *Lus10042068* according to the local Manhattan map and LD heatmap, which has been confirmed to play an important role in rice disease resistance. These large-effect QTLs and candidate genes had great significance in the subsequent breeding for flax PM resistance and in understanding the mechanisms of resistance. Furthermore, we proved that NPQTL was positively correlated with flax PM resistance and had an additive effect. We also showed that the outstanding resistant variety C120 provides the foundation for future breeding of flax disease resistance. Finally, we developed a GS model based on the results of GWAS that may be utilized to direct breeding for PM resistance and increase breeding effectiveness. Nevertheless, GWAS analyses typically only reveal SNPs linked to diseases without elucidating the specific functional connections between these SNPs and the diseases. Therefore, further functional studies are essential to validate the findings of GWAS.

## Data availability statement

The datasets presented in this study can be found in online repositories. The names of the repository/repositories and accession number(s) can be found below: https://www.ncbi.nlm.nih.gov/, PRJNA590636.

## Author contributions

LZ: Data curation, Investigation, Writing – original draft, Writing – review & editing. GL: Investigation, Methodology, Writing – original draft. DG: Methodology, Writing – review & editing. XL: Investigation, Writing – review & editing. MX: Investigation, Writing – original draft. HJ: Writing – review & editing. QY: Investigation, Writing – original draft. FX: Investigation, Writing – original draft. XN: Writing – review & editing. LX: Methodology, Writing – review & editing.

## Supplementary material

Supplementary Figure 1 Average LD decay map of 15 chromosomes in 200 core collections.

Supplementary Figure 2 Repeated detection of 64 resistance loci between different models. M1-M5 correspond to the mrMLM, FASTmrMLM, FASTmrEMMA, pLARmEB, and the ISISEM-BLASSO, respectively.

Supplementary Figure 3 Correlation of NPQTL with DI. Correlation analysis between NPQTL and the DI from 2017 **(A)**, 2019 **(B)**, 2020 **(C)**, 2021 **(D)** and mean **(E)**, **(F)** Correlation analysis between NPQTL and five datasets. **** indicates statistical significance at the 0.01% probability.

Supplementary Figure 4 Four-year resistance identification of Partial resistant materials. **(A)** C120, **(B)** C92, **(C)** C198, **(D)** C123, **(E)** C162, **(F)** C111, **(G)** C193.

## References

[B1] Ahmadi Z. Ghafouri-Kesbi F. Zamani P. (2021). Assessing the performance of a novel method for genomic selection: rrBLUP-method6. J. Genet. 100, 24. doi: 10.1007/s12041-021-01275-5 34187971

[B2] Allaby R. G. Peterson G. W. Merriwether D. A. Fu Y.-B. (2005). Evidence of the domestication history of flax (Linum usitatissimum L.) from genetic diversity of the sad2 locus. Theor. Appl. Genet. 112, 58–65. doi: 10.1007/s00122-005-0103-3 16215731

[B3] Aly A. A. Mansour M. Mohamed H. I. Abd-Elsalam K. A. (2012). Examination of correlations between several biochemical components and powdery mildew resistance of flax cultivars. Plant Pathol. J. 28, 149–155. doi: 10.5423/PPJ.2012.28.2.149

[B4] Arojju S. K. Cao M. Trolove M. Barrett B. A. Inch C. Eady C.. (2020). Multi-trait genomic prediction improves predictive ability for dry matter yield and water-soluble carbohydrates in perennial ryegrass. Front. Plant Sci. 11. doi: 10.3389/fpls.2020.01197 PMC7426495 32849742

[B5] Asgarinia P. Cloutier S. Duguid S. Rashid K. Mirlohi A. Banik M.. (2013). Mapping quantitative trait loci for powdery mildew resistance in flax (Linum usitatissimum L.). Crop Sci. 53, 2462–2472. doi: 10.2135/cropsci2013.05.0298

[B6] Bengtsson T. Åhman I. Manninen O. Reitan L. Christerson T. Due Jensen J.. (2017). A novel QTL for powdery mildew resistance in nordic spring barley (Hordeum vulgare L. ssp. vulgare) revealed by genome-wide association study. Front. Plant Sci. 8. doi: 10.3389/fpls.2017.01954 PMC5694554 29184565

[B7] Chen S. Li Z. Wu G. Jin H. Xie D. Yuan H.. (2018). Screening Pasmo − resistant Germplasm Resources from Flax Varieties. Plant Fiber Sci. China 40, 249–257.

[B8] Covarrubias-Pazaran G. (2016). Genome-assisted prediction of quantitative traits using the R package sommer. PloS One 11, e0156744. doi: 10.1371/journal.pone.0156744 27271781 PMC4894563

[B9] Cui Y. Zhang F. Zhou Y. (2018). The application of multi-locus GWAS for the detection of salt-tolerance loci in rice. Front. Plant Sci. 9. doi: 10.3389/fpls.2018.01464 PMC6180169 30337936

[B10] Dash J. Naik B. S. Mohapatra U. B. (2016). Field screening of linseed genotypes for resistance to powdery mildew (*Oidium lini* Skoric) in the north central plateau zone of Odisha. Int. J. Advanced Res. 4, 961–962. doi: 10.21474/IJAR01

[B11] Dhirhi N. Mehta N. Singh S. (2017). Screening of powdery mildew tolerance in linseed (Linum usitatissimum L.). J. Plant Dev. Sci. 9, 153–156.

[B12] Du X. Huang G. He S. Yang Z. Sun G. Ma X.. (2018). Resequencing of 243 diploid cotton accessions based on an updated A genome identifies the genetic basis of key agronomic traits. Nat. Genet. 50, 796–802. doi: 10.1038/s41588-018-0116-x 29736014

[B13] Dudbridge F. Koeleman B. P. (2004). Efficient computation of significance levels for multiple associations in large studies of correlated data, including genomewide association studies. Am. J. Hum. Genet. 75, 424–435. doi: 10.1086/423738 15266393 PMC1182021

[B14] Duk M. Kanapin A. Rozhmina T. Bankin M. Surkova S. Samsonova A.. (2021). The genetic landscape of fiber flax. Front. Plant Sci. 12. doi: 10.3389/fpls.2021.764612 PMC8691122 34950165

[B15] Fu Y.-B. (2011). Genetic evidence for early flax domestication with capsular dehiscence. Genet. Resour. Crop Evol. 58, 1119–1128. doi: 10.1007/s10722-010-9650-9

[B16] Fu Y.-B. Allaby R. G. (2010). Phylogenetic network of Linum species as revealed by non-coding chloroplast DNA sequences. Genet. Resour. Crop Evol. 57, 667–677. doi: 10.1007/s10722-009-9502-7

[B17] Gu Z. Eils R. Schlesner M. (2016). Complex heatmaps reveal patterns and correlations in multidimensional genomic data. Bioinformatics 32, 2847–2849. doi: 10.1093/bioinformatics/btw313 27207943

[B18] Gunjača J. Carović-Stanko K. Lazarević B. Vidak M. Petek M. Liber Z.. (2021). Genome-Wide association studies of mineral content in common bean. Front. Plant Sci. 12. doi: 10.3389/fpls.2021.636484 PMC7982862 33763096

[B19] Guo D. Jiang H. Yan W. Yang L. Ye J. Wang Y.. (2020). Resequencing 200 flax cultivated accessions identifies candidate genes related to seed size and weight and reveals signatures of artificial selection. Front. Plant Sci. 10. doi: 10.3389/fpls.2019.01682 PMC6976528 32010166

[B20] Hall L. M. Booker H. Siloto R. M. Jhala A. J. Weselake R. J. (2016). “Flax (Linum usitatissimum L.),” in Industrial oil crops (Elsevier), 157–194. doi: 10.1016/B978-1-893997-98-1.00006-3

[B21] He J. Chen G. Li J. Wang J. Yang W. Yang Y.. (2007). Analysis of Field Resistance to Powdery Mildew in Flax Varieties. Plant Fiber Sci. China 29, 141–144.

[B22] He L. Xiao J. Rashid K. Y. Jia G. Li P. Yao Z.. (2019b). Evaluation of genomic prediction for pasmo resistance in flax. Int. J. Mol. Sci. 20, 359. doi: 10.3390/ijms20020359 30654497 PMC6359301

[B23] He L. Xiao J. Rashid K. Yao Z. Li P. Jia G.. (2019a). Genome-wide association studies for pasmo resistance in flax (Linum usitatissimum L.). Front. Plant Sci. 9. doi: 10.3389/fpls.2018.01982 PMC6339956 30693010

[B24] Ikeogu U. N. Akdemir D. Wolfe M. Okeke U. G. Egesi C. N. (2019). Genetic Correlation, Genome-Wide Association and Genomic Prediction of Portable NIRS Predicted Carotenoids in Cassava Roots. Front. Plant Sci. 10. doi: 10.3389/fpls.2019.01570 PMC6904298 31867030

[B25] Javid M. Rosewarne G. M. Sudheesh S. Kant P. Leonforte A. Lombardi M.. (2015). Validation of molecular markers associated with boron tolerance, powdery mildew resistance and salinity tolerance in field peas. Front. Plant Sci. 6. doi: 10.3389/fpls.2015.00917 PMC4621404 26579164

[B26] Jighly A. Lin Z. Pembleton L. W. Cogan N. O. Spangenberg G. C. Hayes B. J.. (2019). Boosting genetic gain in allogamous crops via speed breeding and genomic selection. Front. Plant Sci. 10. doi: 10.3389/fpls.2019.01364 PMC6873660 31803197

[B27] Kang Y. Zhou M. Merry A. Barry K. (2020). Mechanisms of powdery mildew resistance of wheat–a review of molecular breeding. Plant Pathol. 69, 601–617. doi: 10.1111/ppa.13166

[B28] Kuznetsova A. Brockhoff P. B. Christensen R. H. (2017). lmerTest package: tests in linear mixed effects models. J. Stat. software 82, 1–26. doi: 10.18637/jss.v082.i13

[B29] Lan S. Zheng C. Hauck K. McCausland M. Duguid S. D. Booker H. M.. (2020). Genomic prediction accuracy of seven breeding selection traits improved by QTL identification in flax. Int. J. Mol. Sci. 21, 1577. doi: 10.3390/ijms21051577 32106624 PMC7084455

[B30] Li Z. Kemppainen P. Rastas P. Merilä J. (2018). Linkage disequilibrium clustering-based approach for association mapping with tightly linked genomewide data. Mol. Ecol. Resour. 18, 809–824. doi: 10.1111/1755-0998.12893 29673105

[B31] Li D. Liu Q. Schnable P. S. (2021). TWAS results are complementary to and less affected by linkage disequilibrium than GWAS. Plant Physiol. 186, 1800–1811. doi: 10.1093/plphys/kiab161 33823025 PMC8331151

[B32] Li H. Zhang L. Hu J. Zhang F. Chen B. Xu K.. (2017). Genome-wide association mapping reveals the genetic control underlying branch angle in rapeseed (Brassica napus L.). Front. Plant Sci. 8. doi: 10.3389/fpls.2017.01054 PMC5474488 28674549

[B33] Liang Y. Kelemen A. (2008). Statistical advances and challenges for analyzing correlated high dimensional SNP data in genomic study for complex diseases. Stat Surv. 43–60. doi: 10.1214/07-SS026

[B34] Liu W. Maccaferri M. Chen X. Laghetti G. Pignone D. Pumphrey M.. (2017). Genome-wide association mapping reveals a rich genetic architecture of stripe rust resistance loci in emmer wheat (Triticum turgidum ssp. dicoccum). Theor. Appl. Genet. 130, 2249–2270. doi: 10.1007/s00122-017-2957-6 28770301 PMC5641275

[B35] Lorenzana R. E. Bernardo R. (2009). Accuracy of genotypic value predictions for marker-based selection in biparental plant populations. Theor. Appl. Genet. 120, 151–161. doi: 10.1007/s00122-009-1166-3 19841887

[B36] Lou Q. Chen L. Mei H. Wei H. Feng F. Wang P.. (2015). Quantitative trait locus mapping of deep rooting by linkage and association analysis in rice. J. Exp. Bot. 66, 4749–4757. doi: 10.1093/jxb/erv246 26022253 PMC4507776

[B37] Meuwissen T. Hayes B. Goddard M. (2013). Accelerating improvement of livestock with genomic selection. Annu. Rev. Anim. Biosci. 1, 221–237. doi: 10.1146/annurev-animal-031412-103705 25387018

[B38] Pogoda M. Liu F. Douchkov D. Djamei A. Reif J. C. Schweizer P.. (2020). Identification of novel genetic factors underlying the host-pathogen interaction between barley (Hordeum vulgare L.) and powdery mildew (Blumeria graminis f. sp. hordei). PloS One 15, e0235565. doi: 10.1371/journal.pone.0235565 32614894 PMC7332009

[B39] Price A. L. Patterson N. J. Plenge R. M. Weinblatt M. E. Shadick N. A. Reich D. (2006). Principal components analysis corrects for stratification in genome-wide association studies. Nat. Genet. 38, 904–909. doi: 10.1038/ng1847 16862161

[B40] Qiao H. Chen J. (2012). Resistance Evaluation of Flax Varieties to Powdery Mildew. Plant Fiber Sci. China 34, 118–120.

[B41] Rashid K. Duguid S. (2005). Inheritance of resistance to powdery mildew in flax. Can. J. Plant Pathol. 27, 404–409. doi: 10.1080/07060660509507239

[B42] R Core Team (2023). “R: A language and environment for statistical computing,” in R Foundation for Statistical Computing. (Vienna, Austria). Available at: https://www.R-project.org/.

[B43] Remington D. L. Thornsberry J. M. Matsuoka Y. Wilson L. M. Whitt S. R. Doebley J.. (2001). Structure of linkage disequilibrium and phenotypic associations in the maize genome. Proc. Natl. Acad. Sci. 98, 11479–11484. doi: 10.1073/pnas.201394398 11562485 PMC58755

[B44] Robinson G. K. (1991). That BLUP is a good thing: the estimation of random effects. Stat. Sci. 6, 15–32. doi: 10.1214/ss/1177011926

[B45] Singh N. K. Chauhan Y. S. Kumar K. Gupta R. P. (1989). Inheritance of powdery mildew resistance in linseed (Linum usitatissimum L.). Indian J. Genet. Plant Breed. 49, 421–422.

[B46] Speck A. Trouvé J.-P. Enjalbert J. Geffroy V. Joets J. Moreau L. (2022). Genetic architecture of powdery mildew resistance revealed by a genome-wide association study of a worldwide collection of flax (Linum usitatissimum L.). Front. Plant Sci. 13. doi: 10.3389/fpls.2022.871633 PMC9263915 35812909

[B47] Stadlmeier M. Hartl L. Mohler V. (2018). Usefulness of a multiparent advanced generation intercross population with a greatly reduced mating design for genetic studies in winter wheat. Front. Plant Sci. 9. doi: 10.3389/fpls.2018.01825 PMC6291512 30574161

[B48] Sun C. Wang B. Wang X. Hu K. Li K. Li Z.. (2016). Genome-wide association study dissecting the genetic architecture underlying the branch angle trait in rapeseed (Brassica napus L.). Sci. Rep. 6, 33673. doi: 10.1038/srep33673 27646167 PMC5028734

[B49] Tamba C. L. Ni Y.-L. Zhang Y.-M. (2017). Iterative sure independence screening EM-Bayesian LASSO algorithm for multi-locus genome-wide association studies. PloS Comput. Biol. 13, e1005357. doi: 10.1371/journal.pcbi.1005357 28141824 PMC5308866

[B50] Tamba C. L. Zhang Y.-M. (2018). A fast mrMLM algorithm for multi-locus genome-wide association studies. Cold Spring Harbor Laboratory. 341784. doi: 10.1101/341784

[B51] Wang Y. Su J. (2006). Standardization of Description and Data Standards for Flax Germplasm Resources (Beijing: China Agriculture Press).

[B52] Wang W. Ye C. Chen C. Hu G. Ou Q. Zhang J.. (2019). Recent advances of powdery mildew in flax. Plant Fiber Sci. China 41, 478.

[B53] Wen Y.-J. Zhang H. Ni Y.-L. Huang B. Zhang J. Feng J.-Y.. (2018). Methodological implementation of mixed linear models in multi-locus genome-wide association studies. Briefings Bioinf. 19, 700–712. doi: 10.1093/bib/bbw145 PMC6054291 28158525

[B54] Willocquet L. Savary S. Singh K. (2023). Revisiting the use of disease index and of disease scores in plant pathology. Indian Phytopathol. 76, 909–914. doi: 10.1007/s42360-023-00663-4

[B55] Wu J. Zhao Q. Wu G. Zhang S. Jiang T. (2017). Development of novel SSR markers for flax (Linum usitatissimum L.) using reduced-representation genome sequencing. Front. Plant Sci. 7. doi: 10.3389/fpls.2016.02018 PMC5233678 28133461

[B56] Xu S. (2013). Genetic mapping and genomic selection using recombination breakpoint data. Genetics 195, 1103–1115. doi: 10.1534/genetics.113.155309 23979575 PMC3813840

[B57] Xu Y. Liu X. Fu J. Wang H. Wang J. Huang C.. (2020). Enhancing genetic gain through genomic selection: from livestock to plants. Plant Commun. 1, 100005. doi: 10.1016/j.xplc.2019.100005 33404534 PMC7747995

[B58] Yang C. Yu Y. Huang J. Meng F. Pang J. Zhao Q.. (2019). Binding of the Magnaporthe oryzae chitinase MoChia1 by a rice tetratricopeptide repeat protein allows free chitin to trigger immune responses. Plant Cell 31, 172–188. doi: 10.1105/tpc.18.00382 30610168 PMC6391695

[B59] Yang X. Zhao Y. Guan F. Li Z. Liu L. Wu G.. (2008). Genetic analysis of resistance to powdery mildew in flax line 9801–1. Acta Phytopathologica Sin. 38, 656–658.

[B60] Yano K. Yamamoto E. Aya K. Takeuchi H. Lo P.-C. Hu L.. (2016). Genome-wide association study using whole-genome sequencing rapidly identifies new genes influencing agronomic traits in rice. Nat. Genet. 48, 927–934. doi: 10.1038/ng.3596 27322545

[B61] You F. M. Rashid K. Y. Zheng C. Khan N. Li P. Xiao J.. (2022). Insights into the genetic architecture and genomic prediction of powdery mildew resistance in flax (Linum usitatissimum L.). Int. J. Mol. Sci. 23, 4960. doi: 10.3390/ijms23094960 35563347 PMC9104541

[B62] Yu J. Pressoir G. Briggs W. H. Vroh Bi I. Yamasaki M. Doebley J. F.. (2006). A unified mixed-model method for association mapping that accounts for multiple levels of relatedness. Nat. Genet. 38, 203–208. doi: 10.1038/ng1702 16380716

[B63] Zhang Q. (2015). Localization of Resistance Genes against Powdery Mildew in Flax (Harbin: Heilongjiang University).

[B64] Zhang J. Feng J.-Y. Ni Y. Wen Y. Niu Y. Tamba C.. (2017). pLARmEB: integration of least angle regression with empirical Bayes for multilocus genome-wide association studies. Heredity 118, 517–524. doi: 10.1038/hdy.2017.8 28295030 PMC5436030

[B65] Zhang Y. Liu P. Zhang X. Zheng Q. Chen M. Ge F.. (2018). Multi-locus genome-wide association study reveals the genetic architecture of stalk lodging resistance-related traits in maize. Front. Plant Sci. 9. doi: 10.3389/fpls.2018.00611 PMC5949362 29868068

[B66] Zhou Z. (2020). Current Status and Existing Problems of Flaxseed Oil Industry Development in China. China Oils Fats 45, 134–136.

