# Supplementary figures and images for: Genome-wide association study and genomic selection of flax powdery mildew in Xinjiang Province
Source: Front Plant Sci. 2024 May 28;15:1403276. doi: 10.3389/fpls.2024.1403276 (PMC11165360; doi:10.3389/fpls.2024.1403276)

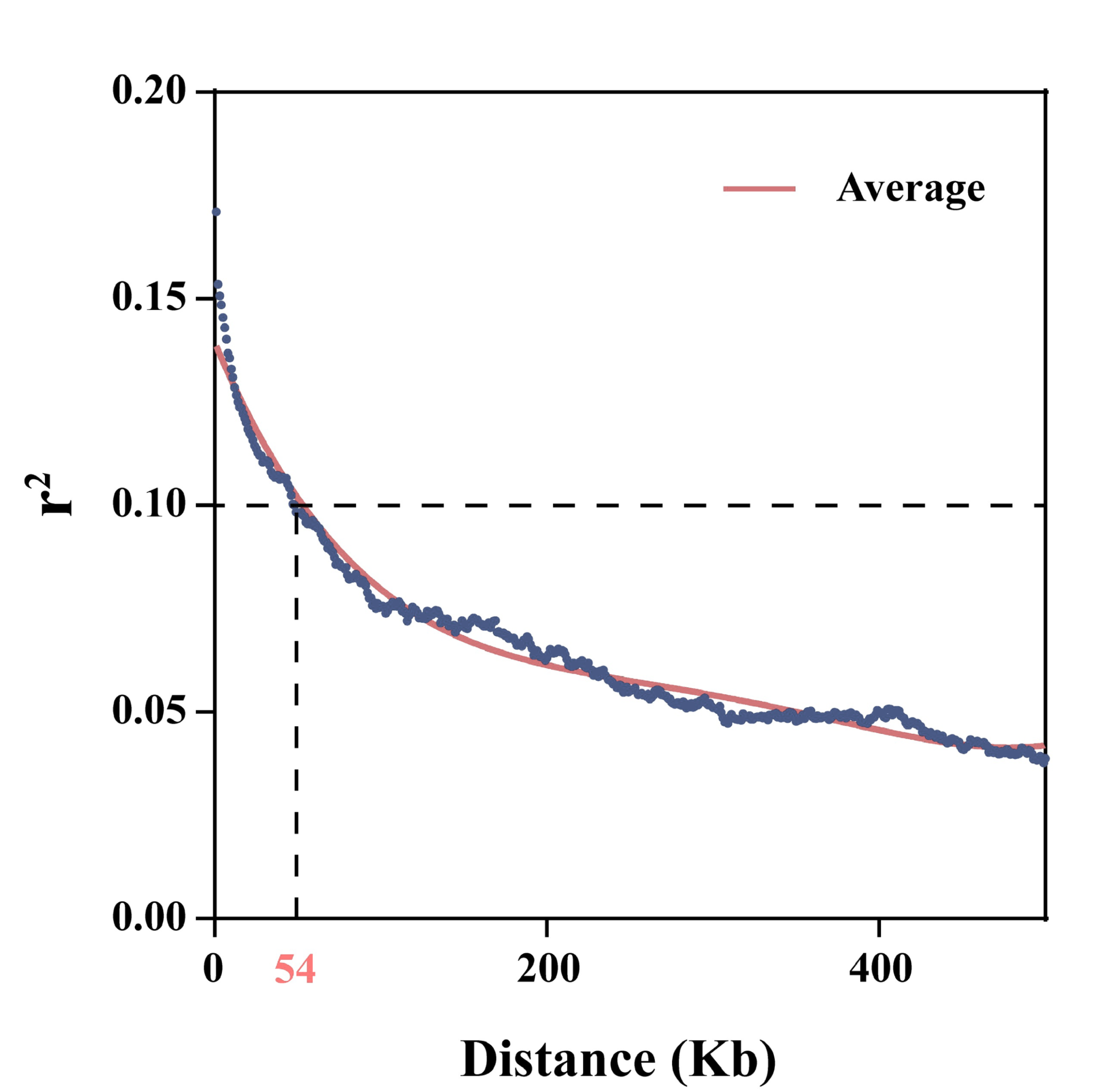

Supplement: Supplementary Figure 1 — Average LD decay map of 15 chromosomes in 200 core collections. [file Image_1.tif]

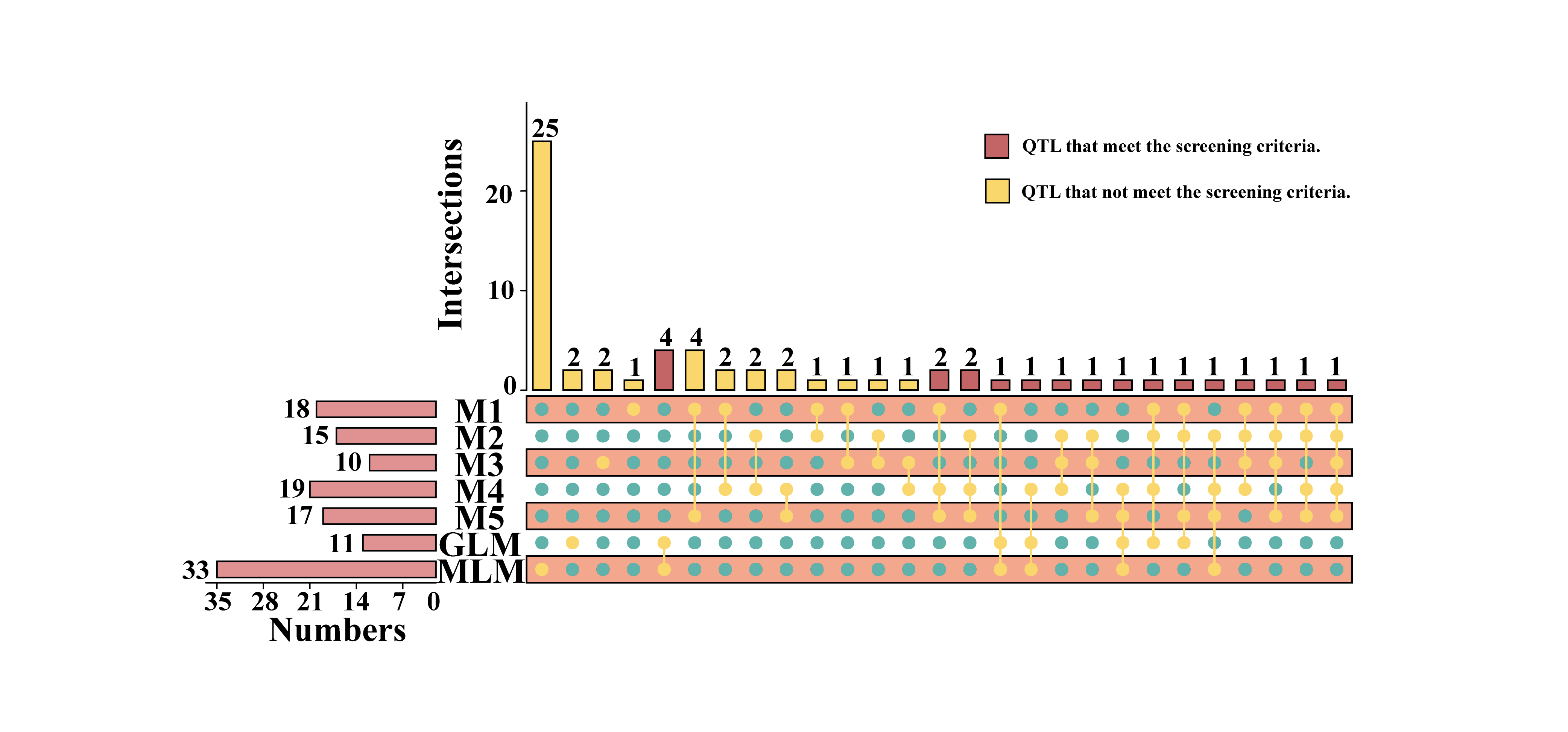

Supplement: Supplementary Figure 2 — Repeated detection of 64 resistance loci between different models. M1-M5 correspond to the mrMLM, FASTmrMLM, FASTmrEMMA, pLARmEB, and the ISISEM-BLASSO, respectively. [file Image_2.tif]

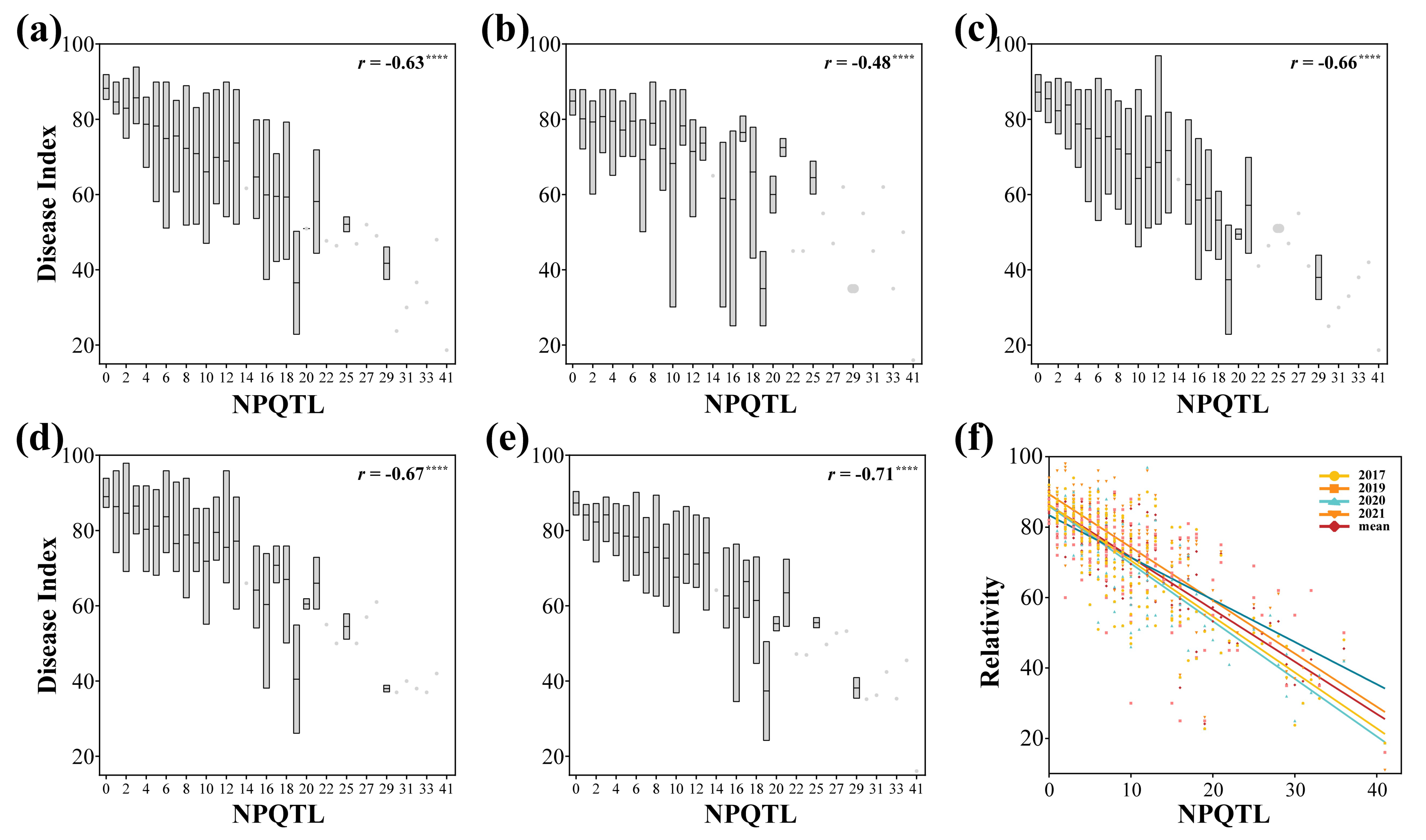

Supplement: Supplementary Figure 3 — Correlation of NPQTL with DI. Correlation analysis between NPQTL and the DI from 2017 (A), 2019 (B), 2020 (C), 2021 (D) and mean (E), (F) Correlation analysis between NPQTL and five datasets. **** indicates statistical significance at the 0.01% probability. [file Image_3.tif]

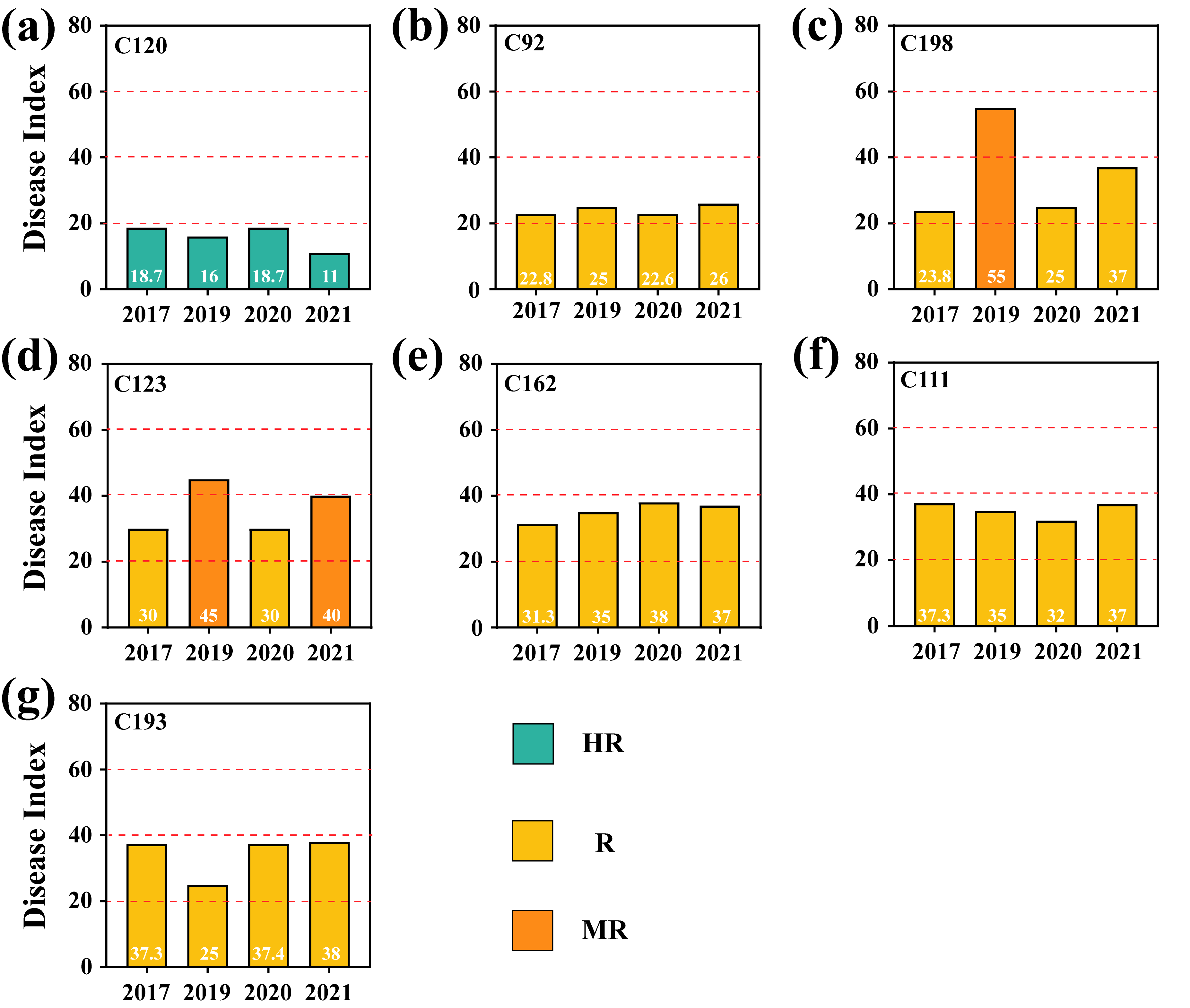

Supplement: Supplementary Figure 4 — Four-year resistance identification of Partial resistant materials. (A) C120, (B) C92, (C) C198, (D) C123, (E) C162, (F) C111, (G) C193. [file Image_4.tif]
