# Supplementary material for: Genome-wide association study and genomic selection of flax powdery mildew in Xinjiang Province
Source: Front Plant Sci. 2024 May 28;15:1403276. doi: 10.3389/fpls.2024.1403276 (PMC11165360; doi:10.3389/fpls.2024.1403276)
Supplement: Supplementary file 5 [file Table_1.doc]

**Table S1 |** 200 parts of flax germplasm source

| Origin | Numbers | Origin | Numbers | Origin | Numbers |
| --- | --- | --- | --- | --- | --- |
| China | 1 | Greece | 1 | Cyprus | 1 |
| United States | 24 | Egypt | 2 | Serbia | 1 |
| Russia | 4 | Iran | 3 | Bolivia | 1 |
| India | 11 | Tajikistan | 3 | Argentina | 5 |
| France | 11 | Japan | 3 | Bulgaria | 1 |
| Canada | 7 | Czechoslovakia | 2 | Mexico | 1 |
| Australia | 3 | Poland | 2 | Zimbabwe | 1 |
| Netherlands | 6 | Pakistan | 1 | Korea | 1 |
| Romanian | 13 | Afghanistan | 1 | Costa Rica | 1 |
| Hungary | 7 | Spain | 2 | Finland | 1 |
| Brazil | 1 | Uruguay | 2 | New Zealand | 1 |
| Morocco | 4 | Turkey | 2 | Ethiopia | 2 |
| Germany | 3 | Indonesia | 1 | Unknown | 61 |
| Belgium | 1 | Italy | 1 |  |  |
