# Supplementary material for: Genome-wide association study and genomic selection of flax powdery mildew in Xinjiang Province
Source: Front Plant Sci. 2024 May 28;15:1403276. doi: 10.3389/fpls.2024.1403276 (PMC11165360; doi:10.3389/fpls.2024.1403276)
Supplement: Supplementary file 7 [file Table_3.doc]

**Table S3** | Disease index variation of 200 accessions.

| **Year** | **Sample Size** | **x ± s** | **Range** | **CV (%)** |
| --- | --- | --- | --- | --- |
| 2017 | 200 | 71.7±15.3 | 18.7-94 | 21.4 |
| 2019 | 200 | 72.3±13.3 | 16-90 | 18.4 |
| 2020 | 200 | 70.9±15.4 | 18.7-97 | 21.7 |
| 2021 | 200 | 75.4±14.1 | 11-98 | 18.6 |
| mean | 200 | 72.6±13.3 | 16.1-90.5 | 18.6 |
