# Supplementary material for: Genome-wide association study and genomic selection of flax powdery mildew in Xinjiang Province
Source: Front Plant Sci. 2024 May 28;15:1403276. doi: 10.3389/fpls.2024.1403276 (PMC11165360; doi:10.3389/fpls.2024.1403276)
Supplement: Supplementary file 8 [file Table_4.doc]

**Table S4** | The average DI of PM resistance in different resistance groups.

| **resistance groups** | **2017** | **2019** | **2020** | **2021** | **Mean** |
| --- | --- | --- | --- | --- | --- |
| HS | 75 | 48 | 71 | 74 | 74 |
| S | 81 | 123 | 80 | 100 | 92 |
| MR | 36 | 21 | 41 | 18 | 27 |
| R | 7 | 7 | 7 | 7 | 6 |
| HR | 1 | 1 | 1 | 1 | 1 |
