# Supplementary material for: Genome-wide association study and genomic selection of flax powdery mildew in Xinjiang Province
Source: Front Plant Sci. 2024 May 28;15:1403276. doi: 10.3389/fpls.2024.1403276 (PMC11165360; doi:10.3389/fpls.2024.1403276)
Supplement: Supplementary file 10 [file Table_6.doc]

**Table S6 |** Number of common QTNs and QTL identified by any two statistical models.

| **Statistical model** | **GLM** | **MLM** | **mrMLM** | **FASTmrMLM** | **FASTmrEMMA** | **pLARmEB** |
| --- | --- | --- | --- | --- | --- | --- |
| MLM | 14 (13) |  |  |  |  |  |
| mrMLM | 4（3） | 3（2） |  |  |  |  |
| FASTmrMLM | 4（2） | 6（5） | 17（15） |  |  |  |
| FASTmrEMMA | 0（0） | 0（0） | 7（6） | 12（10） |  |  |
| pLARmEB | 5（5） | 9（8） | 15（13） | 43（40） | 10（7） |  |
| ISIS EM-BLASSO | 3（1） | 6（5） | 15（13） | 25（23） | 4（3） | 27（26） |
