# Supplementary material for: Genome-wide association study and genomic selection of flax powdery mildew in Xinjiang Province
Source: Front Plant Sci. 2024 May 28;15:1403276. doi: 10.3389/fpls.2024.1403276 (PMC11165360; doi:10.3389/fpls.2024.1403276)
Supplement: Supplementary file 11 [file Table_7.doc]

**Table S7 |** QTNs/QTL for powdery mildew identified using phenotypic data from 4 years and their mean with seven statistical models.

| **Dataset** | **No. of QTL identified** | **No. of QTNs**  **identified** | **Average**  **R^2^ (%)** | **R^2^**  **range (%)** |
| --- | --- | --- | --- | --- |
| 2017 | 76 | 98 | 6.49 | 0.43-18.27 |
| 2019 | 140 | 172 | 11.12 | 0.08-23.64 |
| 2020 | 83 | 91 | 5.21 | 0.21-19.65 |
| 2021 | 112 | 143 | 10.81 | 0.24-21.85 |
| Mean | 114 | 157 | 9.84 | 0.12-21.75 |
