# Supplementary material for: Genome-wide association study and genomic selection of flax powdery mildew in Xinjiang Province
Source: Front Plant Sci. 2024 May 28;15:1403276. doi: 10.3389/fpls.2024.1403276 (PMC11165360; doi:10.3389/fpls.2024.1403276)
Supplement: Supplementary file 12 [file Table_8.doc]

**Table S8 |** Number of common QTNs/QTL identified from any two datasets.

| **Dataset** | **2017** | **2019** | **2020** | **2021** |
| --- | --- | --- | --- | --- |
| 2019 | 7（5） |  |  |  |
| 2020 | 32（25） | 8（5） |  |  |
| 2021 | 18（9） | 50（31） | 16（11） |  |
| Mean | 41（28） | 57（34） | 34（25） | 77（47） |
