# Supplementary material for: Genome-wide association study and genomic selection of flax powdery mildew in Xinjiang Province
Source: Front Plant Sci. 2024 May 28;15:1403276. doi: 10.3389/fpls.2024.1403276 (PMC11165360; doi:10.3389/fpls.2024.1403276)
Supplement: Supplementary file 14 [file Table_10.doc]

**Table S10 | Statistics on agronomic characters of Partial resistant materials.**

| **Code** | **Name** | **Type of resistance** | **Origin** | **Plant height**  **(cm)** | **Stem diameter**  **(mm)** | **Capsule number** | **1000-seed weight**  **(g)** | **Stem yield**  **(kg·hm^-2^)** | **Seed yield**  **(kg·hm^-2^)** |
| --- | --- | --- | --- | --- | --- | --- | --- | --- | --- |
| C120 | Q0415 | HR | Belgium | 86.94 | 2.55 | 51.06 | 4.21 | 8611 | 1625 |
| C92 | RABA 0189 | R | Poland | 96.50 | 2.98 | 51.00 | 6.02 | 6455 | 1413 |
| C162 | CIli 1832 | R | India | 37.54 | 1.45 | 26.43 | 5.58 | 833 | 360 |
| C111 | CIli 1924 | R | Egypt New | 73.21 | 2.21 | 46.89 | 4.59 | 2777 | 1104 |
| C193 | HINU | R | Zealand | 66.50 | 2.42 | 46.72 | 5.14 | 4166 | 1958 |
